# Adverse effects of COVID-19 vaccines and measures to prevent them

**DOI:** 10.1186/s12985-022-01831-0

**Published:** 2022-06-05

**Authors:** Kenji Yamamoto

**Affiliations:** Department of Cardiovascular Surgery, Center of Varicose Veins, Okamura Memorial Hospital, 293-1 Kakita Shimizu-cho, Sunto-gun, Shizuoka, 411-0904 Japan

**Keywords:** COVID-19, Risk factor, Critically ill patients, Vaccination, Vaccine-acquired immunodeficiency syndrome

## Abstract

Recently, *The Lancet* published a study on the effectiveness of COVID-19 vaccines and the waning of immunity with time. The study showed that immune function among vaccinated individuals 8 months after the administration of two doses of COVID-19 vaccine was lower than that among the unvaccinated individuals. According to European Medicines Agency recommendations, frequent COVID-19 booster shots could adversely affect the immune response and may not be feasible. The decrease in immunity can be caused by several factors such as N1-methylpseudouridine, the spike protein, lipid nanoparticles, antibody-dependent enhancement, and the original antigenic stimulus. These clinical alterations may explain the association reported between COVID-19 vaccination and shingles. As a safety measure, further booster vaccinations should be discontinued. In addition, the date of vaccination should be recorded in the medical record of patients. Several practical measures to prevent a decrease in immunity have been reported. These include limiting the use of non-steroidal anti-inflammatory drugs, including acetaminophen to maintain deep body temperature, appropriate use of antibiotics, smoking cessation, stress control, and limiting the use of lipid emulsions, including propofol, which may cause perioperative immunosuppression. In conclusion, COVID-19 vaccination is a major risk factor for infections in critically ill patients.

**Dear Editor,**

The coronavirus disease (COVID-19) pandemic has led to the widespread use of genetic vaccines, including mRNA and viral vector vaccines. In addition, booster vaccines have been used, but their effectiveness against the highly mutated spike protein of Omicron strains is limited. Recently, *The Lancet* published a study on the effectiveness of COVID-19 vaccines and the waning of immunity with time [1]. The study showed that immune function among vaccinated individuals 8 months after the administration of two doses of COVID-19 vaccine was lower than that among unvaccinated individuals. These findings were more pronounced in older adults and individuals with pre-existing conditions. According to the European Medicines Agency’s recommendations, frequent COVID-19 booster shots could adversely affect the immune response and may not be feasible [2]. Several countries, including Israel, Chile, and Sweden, are offering the fourth dose to only older adults and other groups rather than to all individuals [3].

The decrease in immunity is caused by several factors. First, N1-methylpseudouridine is used as a substitute for uracil in the genetic code. The modified protein may induce the activation of regulatory T cells, resulting in decreased cellular immunity [4]. Thereby, the spike proteins do not immediately decay following the administration of mRNA vaccines. The spike proteins present on exosomes circulate throughout the body for more than 4 months [5]. In addition, in vivo studies have shown that lipid nanoparticles (LNPs) accumulate in the liver, spleen, adrenal glands, and ovaries [6], and that LNP-encapsulated mRNA is highly inflammatory [7]. Newly generated antibodies of the spike protein damage the cells and tissues that are primed to produce spike proteins [8], and vascular endothelial cells are damaged by spike proteins in the bloodstream [9]; this may damage the immune system organs such as the adrenal gland. Additionally, antibody-dependent enhancement may occur, wherein infection-enhancing antibodies attenuate the effect of neutralizing antibodies in preventing infection [10]. The original antigenic sin [11], that is, the residual immune memory of the Wuhan-type vaccine may prevent the vaccine from being sufficiently effective against variant strains. These mechanisms may also be involved in the exacerbation of COVID-19.

Some studies suggest a link between COVID-19 vaccines and reactivation of the virus that causes shingles [12, 13]. This condition is sometimes referred to as vaccine-acquired immunodeficiency syndrome [14]. Since December 2021, besides COVID-19, Department of Cardiovascular Surgery, Okamura Memorial Hospital, Shizuoka, Japan (hereinafter referred to as “the institute”) has encountered cases of infections that are difficult to control. For example, there were several cases of suspected infections due to inflammation after open-heart surgery, which could not be controlled even after several weeks of use of multiple antibiotics. The patients showed signs of being immunocompromised, and there were a few deaths. The risk of infection may increase. Various medical algorithms for evaluating postoperative prognosis may have to be revised in the future. The media have so far concealed the adverse events of vaccine administration, such as vaccine-induced immune thrombotic thrombocytopenia (VITT), owing to biased propaganda. The institute encounters many cases in which this cause is recognized. These situations have occurred in waves; however, they are yet to be resolved despite the measures implemented to routinely screen patients admitted for surgery for heparin-induced thrombocytopenia (HIT) antibodies. Four HIT antibody-positive cases have been confirmed at the institute since the start of vaccination; this frequency of HIT antibody-positive cases has rarely been observed before. Fatal cases due to VITT following the administration of COVID-19 vaccines have also been reported [15].

As a safety measure, further booster vaccinations should be discontinued. In addition, the date of vaccination and the time since the last vaccination should be recorded in the medical record of patients. Owing to the lack of awareness of this disease group among physicians and general public in Japan, a history of COVID-19 vaccination is often not documented, as it is in the case of influenza vaccination. The time elapsed since the last COVID-19 vaccination may need to be considered when invasive procedures are required. Several practical measures that can be implemented to prevent a decrease in immunity have been reported [16]. These include limiting the use of non-steroidal anti-inflammatory drugs, including acetaminophen, to maintain deep body temperature, appropriate use of antibiotics, smoking cessation, stress control, and limiting the use of lipid emulsions, including propofol, which may cause perioperative immunosuppression [17].

To date, when comparing the advantages and disadvantages of mRNA vaccines, vaccination has been commonly recommended. As the COVID-19 pandemic becomes better controlled, vaccine sequelae are likely to become more apparent. It has been hypothesized that there will be an increase in cardiovascular diseases, especially acute coronary syndromes, caused by the spike proteins in genetic vaccines [18, 19]. Besides the risk of infections owing to lowered immune functions, there is a possible risk of unknown organ damage caused by the vaccine that has remained hidden without apparent clinical presentations, mainly in the circulatory system. Therefore, careful risk assessments prior to surgery and invasive medical procedures are essential. Randomized controlled trials are further needed to confirm these clinical observations.

In conclusion, COVID-19 vaccination is a major risk factor for infections in critically ill patients.

## Data Availability

Not applicable.

## Abbreviations

COVID-19: Coronavirus disease 2019
HIT: Heparin-induced thrombocytopenia
LPN: Lipid nanoparticle
VITT: Vaccine-induced immune thrombotic thrombocytopenia
